# Supplementary material for: Creating boundaries along a synthetic frequency dimension
Source: Nat Commun. 2022 Jun 13;13:3377. doi: 10.1038/s41467-022-31140-7 (PMC9192711; doi:10.1038/s41467-022-31140-7)
Supplement: Supplementary file 1 — Supplementary Information [file 41467_2022_31140_MOESM1_ESM.pdf]

Supplementary Information for "Creating boundaries along a synthetic frequency dimension"

Avik Dutt,[1,2] Luqi Yuan,[3] Ki Youl Yang,[1] Kai Wang,[1]
Siddharth Buddhiraju,[1] Jelena Vučković,[1] and Shanhui Fan[1,*]

[1]*Ginzton Laboratory and Department of Electrical Engineering, Stanford University, Stanford, CA 94305, USA*

[2]*Department of Mechanical Engineering, and Institute for Physical Science and Technology, University of Maryland, College Park, MD 20742, USA*

[3]*State Key Laboratory of Advanced Optical Communication Systems and Networks, School of Physics and Astronomy, Shanghai Jiao Tong University, Shanghai 200240, China*

[*] shanhui@stanford.edu

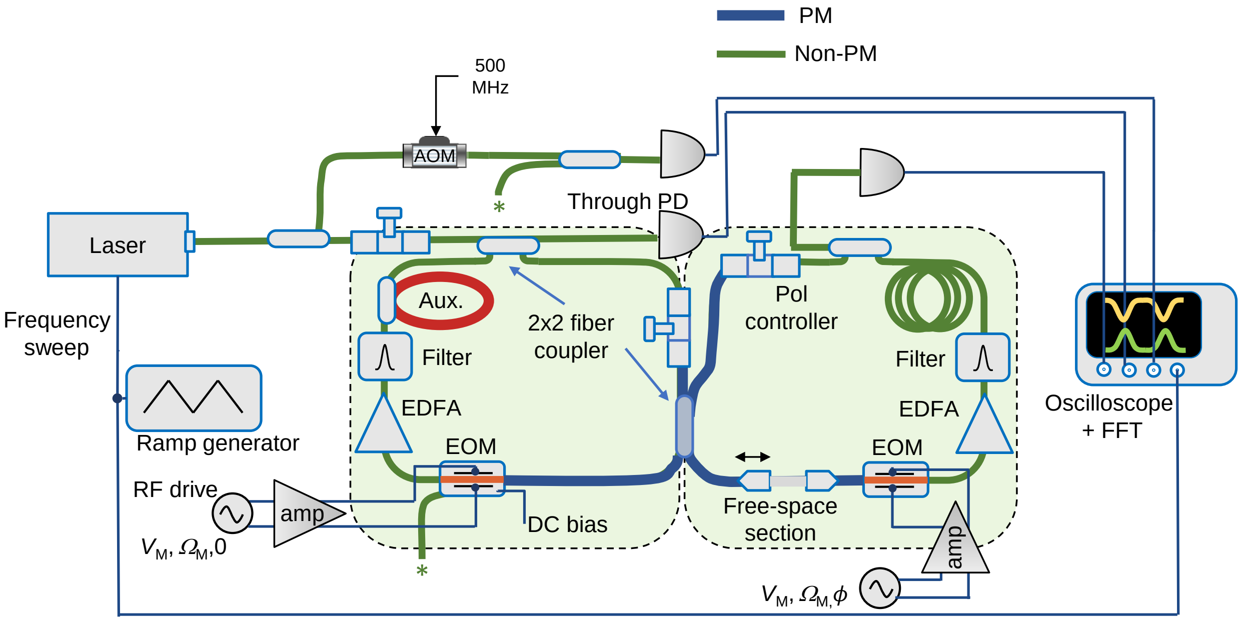

Figure S1. Detailed experimental setup. EOM: electro-optic modulator. AOM: acousto-optic modulator. Aux: auxiliary ring resonator. PD: Photodiode. amp: RF amplifier. PM: polarization-maintaining. EDFA: erbium doped fiber amplifier.

I. CHANGE IN FSR DUE TO AUXILIARY RING

As stated in the main text, the inclusion of an auxiliary ring resonator with a length L_a much smaller than the main cavity length L_0, creates a boundary every $N = L_0/L_a$ modes. This is due to the strong perturbation of the main cavity resonance positions for the main cavity modes that are spectrally aligned with the auxiliary ring modes [Fig. 1]. Besides the creation of boundaries at the on-resonance coupled modes, the auxiliary ring also induces a small off-resonance change in the free-spectral range (FSR) of the main cavity. Such a shift has to be accounted for in experiments by reducing the modulation frequency to match the modified FSR. We quantitatively study the change in FSR in this section.

Let us first look at the phase response seen at the through port of the auxiliary ring resonator [Fig. S2(a)]. This through port is part of the main cavity. The phase response shows sharp jumps of 2π near the auxiliary ring's resonances. Near the anti-resonance at ω_0, the phase varies approximately linearly with frequency, as shown by the inset of Fig. S2(a). The slope of this line is related to the increase in FSR due to the auxiliary ring. We plot this slope using solid lines in Fig. S2(b) over several FSRs of the main cavity.

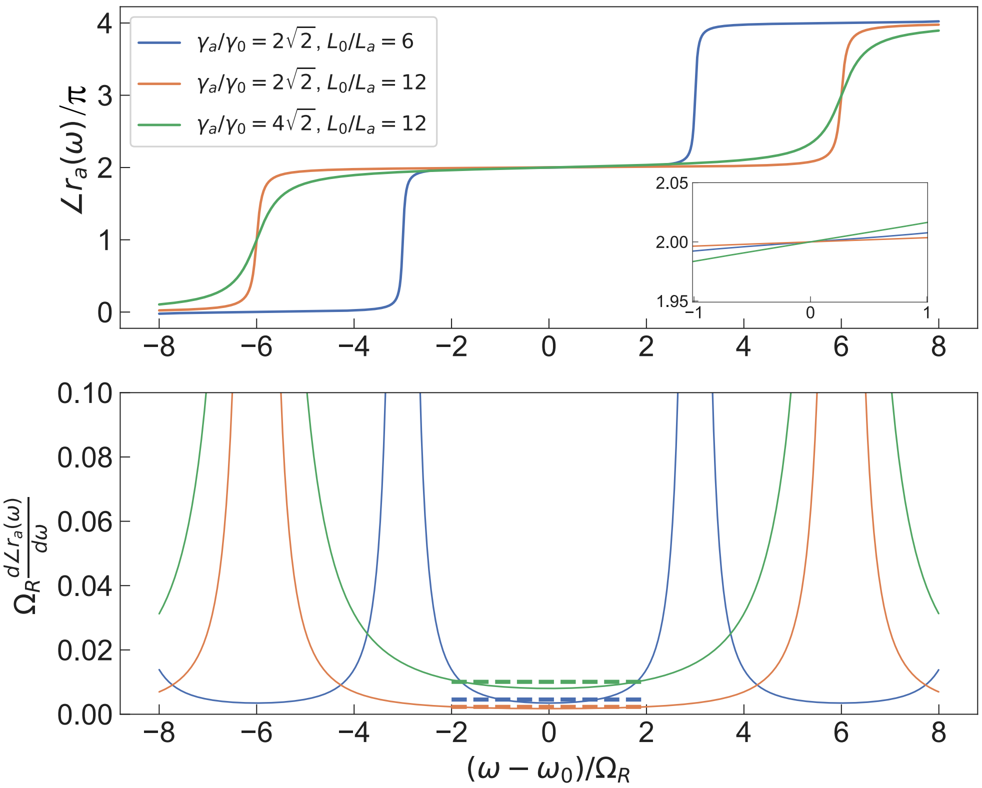

Figure S2. Off-resonance change in free-spectral range (FSR) due to auxiliary ring. **(a)** Phase response of the auxiliary ring resonator for various evanescent couplings γ_a/γ_0, and for various lengths L_a/L_0. The inset shows a zoom-in around anti-resonance at ω_0. Near anti-resonance the phase variation is approximately linear with frequency, and this linear slope can be used to estimate the change in FSR. **(b)** Frequency derivative of the phase response (solid lines). The dashed lines are analytical estimates of δFSR for parameters in the legend of (a) using Eq.(S2). We ignore intrinsic loss in the auxiliary ring ($a = 1$ in Eq. (S2)) to focus on the FSR change due to its phase response, especially since the dependence of δFSR on a is weak. As expected, δFSR increases with an increase in γ_a and L_a. Note that Ω_R is the FSR of the main cavity.

Analytically, the fractional change in the effective FSR to the lowest order can be modeled by the frequency derivative of the phase shift induced by the auxiliary ring at anti-resonance (i.e. $\beta(\omega)L_a = (2m+1)\pi,\ m \in \mathcal{Z}$):

$$\delta\text{FSR} \approx \Omega_R \frac{d}{d\omega} \angle r_a(\omega)\big|_{\beta(\omega)L_a=(2m+1)\pi} \tag{S1}$$

$$\approx \frac{L_a}{L_0} \cdot \frac{a\gamma_a^2}{a + (1+a^2)\sqrt{1-\gamma_a^2}} \tag{S2}$$

where $a = \exp(-\alpha_a L_a/2)$ is the roundtrip transmission amplitude of the auxiliary ring for a propagation loss α_a, and γ_a is the splitting amplitude at the evanescent coupler connecting the two rings. As expected, this decrease in the FSR increases with γ_a and with a reduction in the size of the finite lattice $N = L_0/L_a$. The lowest order analytical estimate from Eq. (S2) is plotted as horizontal dashed lines in Fig. S2(b), and it agrees reasonably well with the solid lines.

II. DISCRETIZED BAND STRUCTURE

In this section we show that the discrete band structures from Fig. 3 correspond to finite lattices with open boundary conditions (OBCs) and not to periodic boundary conditions (PBCs).

In general, the eigenspectrum of a Hermitian Hamiltonian with a finite number of lattice sites N approximates the band structure of the infinite system well for $N \to \infty$ [Fig. S3(a) for $N = 80$]. This is true whether PBCs or OBCs are used. On the other hand, for a reasonably small N, the eigenspectrum with periodic boundary conditions discretely samples the Bloch band structure of the infinite system with good accuracy. However, the eigenspectrum with open boundary conditions deviates from the Bloch bands significantly for reasonably small N [Fig. S3(b) for $N = 8$]. Since both OBCs and PBCs lead to discrete band structures, in Fig. S3(c) and (d) we compare the simulated time-resolved transmission as in Fig. 4 with the eigenspectrum of panel (b). We see that the colormap has peaks at energies (or equivalently $\Delta\omega$) that correspond to the OBC eigenspectrum very well, and not the PBC spectrum. The broadening of the peaks can be attributed to the finite linewidths of the cavity modes. These simulations further corroborate the conclusions in the main text that the auxiliary ring creates open boundaries.

III. THEORY AND SIMULATION APPROACHES

In this section we provide pointers to the theory and simulation techniques that are used throughout the article. The primary techniques used are the tight-binding model (TBM) and the Floquet scattering matrix model. The TBM describes an idealized lattice, without taking into account how the lattice is implemented. Our TBM also does not take into account

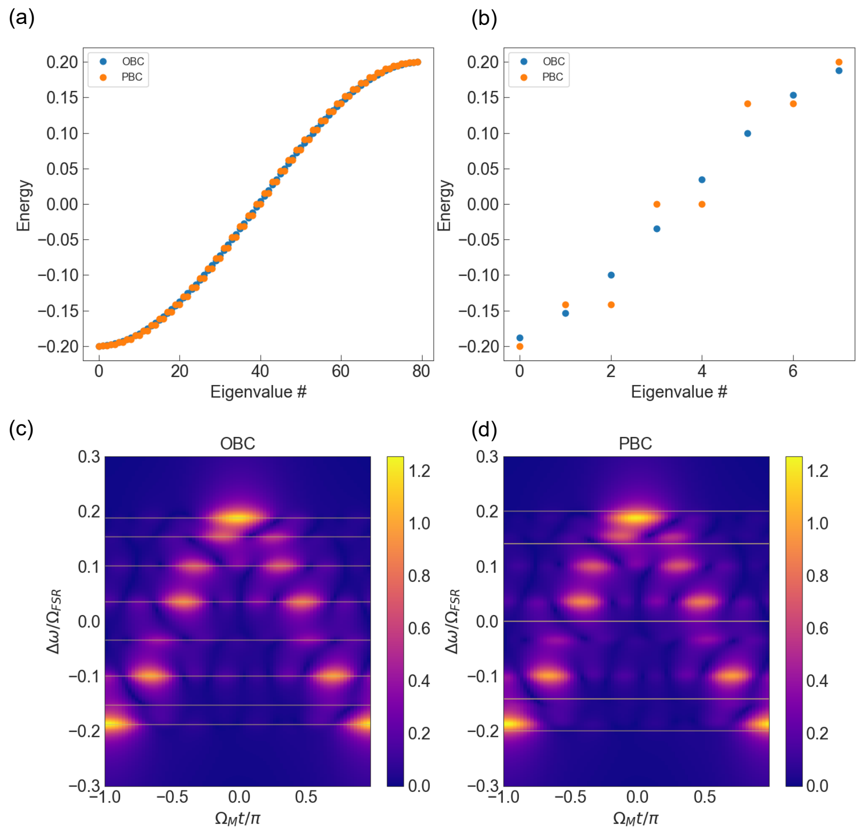

Figure S3. Comparison of eigenspectrum with periodic boundary conditions (PBCs) and open boundary conditions (OBCs). **(a)** Eigenspectrum with $N = 80$. The PBC and OBC spectra agree reasonably well. **(b)** Eigenspectrum with $N = 8$. The PBC and PBC differ significantly. These spectra were calculated from a tight-binding model Hamiltonian with nearest neighbor coupling $J = 0.1$. **(c), (d)** Colormaps show the simulated time-resolved transmission based on a Floquet analysis for $N = 8$. The horizontal lines are overlays of the eigenspectrum from (b) for OBC (c) and PBC (d). The location of peaks in the time-resolved transmission agrees with the OBC spectrum but not the PBC spectrum.

the periodically driven nature of the system. The Floquet scattering matrix analysis, on the other hand, explicitly considers the time-periodic modulation applied to the system. It also considers the field variation at various discrete points that make up the system, such as the modulator, the evanescent couplers and so on. Both these techniques are described in depth in our recent tutorial article, Ref. [1]. The Floquet scattering matrix method is also discussed in the supplementary information of Refs. [2–4].

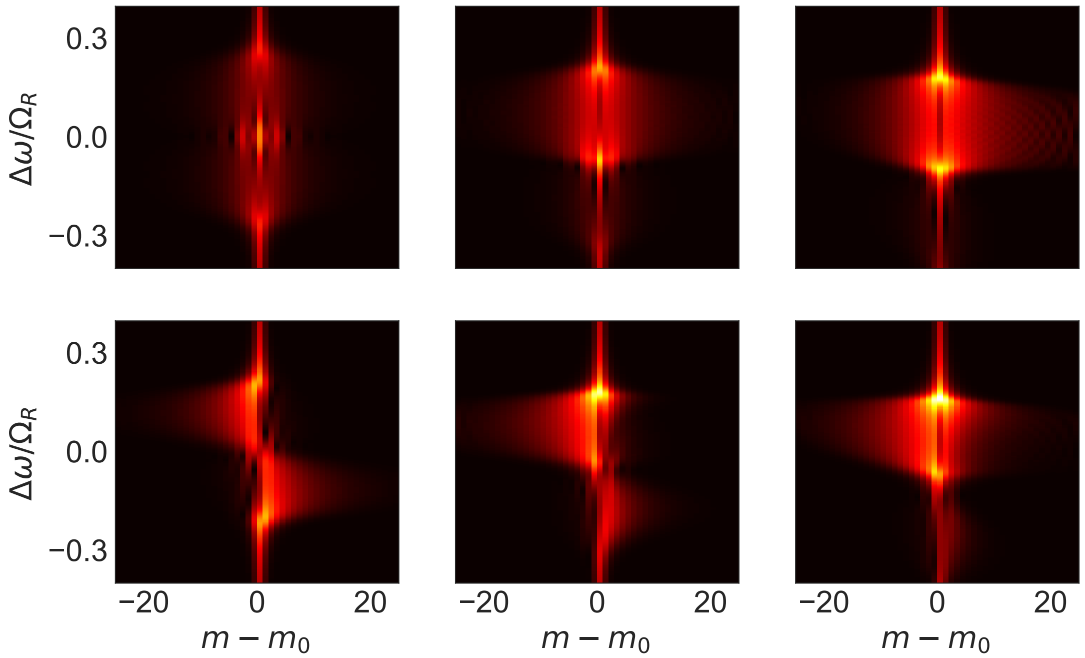

Figure S4. Frequency lattice occupation in the excited (left) ring, similar to Fig. 5 (e) and (i) based on Floquet scattering matrix simulations. Top row: trivial regime ($\phi = 0$). Bottom row: topological regime ($\phi = \pi/2$). The misalignment in the ring lengths increases from left to right, going from 0% (left) to 0.1% (center) to 1% (right). Light transport is disrupted both in the topological and the trivial regime due to mismatch in ring lengths.

IV. SENSITIVITY TO RING-LENGTH VARIATION

While light transport in the synthetic quantum Hall ladder is robust against the presence of a boundary, it is sensitive to the mismatch in the lengths of the two rings. Intuitively, a difference in the ring lengths would make the frequency modes drift from each other. If the frequency drift over a few modes is more than approximately coupling rate between the rings, the equivalent lattice picture in Fig. 5 breaks down. For simplicity, we consider an unbounded lattice in frequency space with the parameters $\gamma_{12}/\gamma_0 = 3, \gamma_a = 0, J/\Omega_R = 0.07$. As can be seen from Fig. S4, this is true both for the topological and the trivial regime. Note that a 0.1% difference in ring lengths is quite large (4 cm in our case).

[1] Yuan, L., Dutt, A. & Fan, S. Synthetic frequency dimensions in dynamically modulated ring resonators. *APL Photonics* **6**, 071102 (2021).

[2] Dutt, A. *et al.* A single photonic cavity with two independent physical synthetic dimensions. *Science* **367**, 59–64 (2020).

[3] Leefmans, C. *et al.* Topological dissipation in a time-multiplexed photonic resonator network. *Nat. Phys.* (2022).

[4] Buddhiraju, S., Dutt, A., Minkov, M., Williamson, I. A. D. & Fan, S. Arbitrary linear transformations for photons in the frequency synthetic dimension. *Nature Communications* **12**, 2401 (2021).
